# Strategies for General Surgery Training Programs During the COVID-19 Pandemic

**DOI:** 10.1177/0003134820966271

**Published:** 2020-11

**Authors:** Alyse M. E. Ragauskas, Anthony M. Scott, Dudley B. Christie, Danny M. Vaughn, Amy B. Christie, Dennis W. Ashley

**Affiliations:** 1 12241 Mercer University School of Medicine, Macon, GA, USA; 2 Department of Surgery, 5223 The Medical Center Navicent Health, Macon, GA, USA

**Keywords:** surgical education, special topics, resident education, COVID-19, critical care

## Abstract

The COVID-19 pandemic presented a unique challenge for Medical systems worldwide. Initial response to the crisis situation for the pandemic closely mirrored plans for a mass casualty event. By leveraging resources including human and physical, and by dividing our surgeon workforce into micro teams we were able to create a flexible and responsive infrastructure to address the crisis as it unfolded. By adoption of virtual platforms and equal division of labor, surgical resident education was continued. Specific adjustments to the schedule and curriculum for medical students allowed them to continue their studies safely and on schedule. Our model serves as an example by which hospital systems of similar size may utilize principles of mass casualty preparedness to craft their own plan for a future contagion response strategy.

## Introduction

In early 2020, the novel coronavirus (COVID-19) created a health care delivery challenge that has never been seen by any modern care delivery infrastructure. As clinicians and researchers race to better understand the virus, its biology, and seek effective treatment methods, assumptions must align with the possibility that the demands on our health care systems may only escalate. The virus-related mortality count continues to rise; the total number of infections, as well as the resultant morbidity, may never be fully known and health care systems on a global scale have been strained to crisis level magnitudes. Mass casualty training has provided hospital systems a framework of experience and parallel processes in terms of resource utilization, closed communication loops, supply chain assessment, and patient triage strategies. However, the acutely injured patient surge seen with mass casualties is distinct from the acutely ill and highly infectious patient surge as the latter places the workforce at risk. While the difficulties of meeting the demands of COVID-19-related patient surges include, but are not limited to, access to ventilators, lack of intensive care unit (ICU) bed availability, and shortages of personal protective equipment (PPE), an additional concern is maintaining and training a healthy workforce.

Graduate medical education programs have played a central role in managing the influx of COVID-19 patients while balancing the expected volume of acute and chronic disease admissions in hospitals on a national scale.^1,2^ Residents and faculty of all specialties have united as a workforce to provide care during the course of the pandemic through a variety of surge plans, many of which are unique to their individual institutions size, need, and capacity. However, the rapidity with which COVID-19 engulfed health care systems, coupled with its highly infectious potential, precluded the opportunity for many institutions to plan, organize, and prepare. While high-quality patient care is a priority, mitigating infectious risk to the workforce is paramount in order to maintain a workforce that is able to safely deliver the care needed.

The Medical Center, Navicent Health is an academic tertiary referral center and an American College of Surgeons Verified Level I Trauma Center serving Central and South Georgia. Our Department of Surgery is a 5-year general surgery residency with 5 residents per tier and 2 fellows in our surgical critical care fellowship. Additionally, The Medical Center serves as the primary teaching hospital for Mercer University School of Medicine hosting student clerkships and electives year-round. The importance of preparation and planning became evident as reports of the highly infectious nature of COVID-19 came into global awareness in early 2020. As we braced for our surge of COVID-19-related cases, we developed an operational strategy to maintain patient care teams while limiting risk and exposure of our residents and staff. Through insulating our care delivery groups, our aim was to avoid illness and therefore serve patients without a critical workforce shortage. The purpose of this article is to describe our surge model and experience within our Department of Surgery as we attempted to balance patient care responsibilities and educational endeavors while mitigating COVID-19 exposure and infection risk.

## Department of Surgery Overview

The Department of Surgery is typically staffed by 7 full-time general surgeons, 4 of which are also board-certified in critical care, 2 dedicated intensivists, 6 advanced practice professionals, 2 critical care fellows, and 25 residents. Under normal circumstances, service lines include 4 general and acute care surgery teams, individual teams for pediatric surgery, surgical oncology and colorectal surgery, trauma, critical care, vascular surgery, and a night float system. Additionally, 1 to 2 residents are away for 5 weeks at a time for rural surgery, transplant, and/or research.

## Department of Surgery Preparation

Anticipating an initial need for isolation and treatment of COVID-19 patients, 2 high capacity ICUs and 1 large inpatient ward were designated for patients under investigation and COVID positive patients only in early March 2020. With a surge imminent and the need for expanded critical care services expected, the hospital’s incident command team identified patient care areas within the hospital that could be readily adapted to provide necessary physical resources to care for critically ill patient populations. The Department of Surgery faculty met and a task force was assembled to develop a deployment strategy for personnel considering the factors in Table 1. All residents and staff were educated in regard to proper PPE use and disposal. N95 masks, face shields, and hand sanitizer were made available to all residents and attendings. Additionally, surgical critical care faculty who had served in the COVID isolation ward already provided virtual didactic sessions with regard to contemporary treatment guidelines and management strategies. These sessions were mandatory for all residents and open to all providers within the department.

**Table 1.** Factors Considered in Team Restructuring.

Continuity of care on essential service lines
Limit interpersonal interactions on daily basis to prevent cross contamination
Allow for time off between shifts to allow for recovery and comply with ACGME duty hour restrictions
Create appropriately sized teams to allow for reserve capacity and redundancy
Adopt a culture of availability while at home
Flexibility to allow a rapid expansion and de-escalation of in-house personnel as needed
Temporary changes to ACGME and ABS case requirements
Professional society recommendations regarding performance of elective/urgent/emergent procedures
Appropriate distribution of experience across service lines, evaluating both experience and capacity
Continue to provide high quality educational opportunities for trainees

Abbreviation: ACGME, Accreditation Council for Graduate Medical Education; ABS, American Board of Surgery.

### Surgical Case Scheduling

The department immediately adopted the recommendation made by multiple national and regional professional societies to postpone elective cases in order to preserve PPE. Surgical cases were designated as emergent or time sensitive. No purely elective cases were scheduled. Outpatient, time-sensitive surgical procedures were limited to diagnoses where pain, malignancy, or predictable, impending patient harm were identified.

### Ambulatory Clinic Adjustments

Ambulatory clinic volumes and patient traffic were carefully scrutinized. In an effort to limit patient congestion in office spaces, a department wide conversion to telehealth platforms was adopted. All patients scheduled for office encounters were reviewed with the attending surgeon and their nursing lead on a weekly basis. Patients amenable to virtual or telehealth visits were triaged into 1 clinic period per week for each attending surgeon. Patients in need of a physical visit were also identified. Clinic days for patient encounters were consolidated to where no more than 3 office days would be required per week, allowing the office staff to be reduced to essential providers and their support staff only. The patients were scheduled to avoid the risk of waiting room congestion, screened for infectious risk factors prior to entry, and never allowed in the same space as another patient once inside our office.

### Surgical Micro-teams

In order to rapidly decrease in-house personnel and expand reserve coverage, the general surgery service lines were restructured into 7 micro-teams to consist of a single attending along with 1 senior and 1 junior resident (Table 2). These individual teams then were in turn responsible for in-house duties associated with an assigned twenty-four-hour call shift following the monthly published attending call schedule. In-house duties consisted of inpatient rounds on all general surgery inpatients, all new patient consults including floor and emergency center (EC), all new patient trauma consults, trauma code activations, and assistance with bedside rounding responsibilities the following morning. At the completion of each call shift, the exiting team would provide a detailed handoff to the oncoming team through a virtual platform. Members of each of these teams were asked to socially distance when not in house and to avoid contact with members of other micro-teams to avoid “cross contamination” risks. If 1 team were to become exposed and required to quarantine, there were additional teams available to fill the patient care gap. The chief residents of all general surgery services met virtually to review the inpatient census and operating room case schedule regularly to identify foreseeable needs and organize coverage in a timely fashion. The on-call and post-call teams met each morning to review new admissions or consultations and assign bedside rounding responsibilities as appropriate. The on-call attending each day was responsible for rounds on all general surgery inpatients that day in addition to all new admissions or consultations for a 24-hour period to include general surgery and trauma.

**Table 2.** Team Structure and Resident Assignment.

General Surgery Service	Attending	Associated resident	
	A^a^	PGY4	PGY2
	B^a^	PGY5	PGY2
	C^a^	PGY4	PGY2
	D^a^	PGY4	PGY3
	E	PGY5	PGY1
	F	PGY5	PGY1
		G	PGY4	PGY3

Subspecialty services	Service	Associated resident	
	Trauma floor	PGY4	PGY1
	STICU	PGY2	
	Pediatric surgery	PGY3	
	Surgical oncology/colorectal surgery	PGY3	
	Vascular surgery	PGY5	
		Night float	PGY2	PGY2

Abbreviations: PGY, Postgraduate year; STICU, Surgical Trauma Intensive Care Unit.

a Attending board-certified in critical care.

### Micro-team Assignments

Surgical case coverage was addressed as follows: Inpatient and acute care surgery cases were covered by the on-call attending and residents on the day and time for which they were scheduled. The limited outpatient procedures were covered by the attending and assigned team residents. In those cases, calling for a unique skill set, the consulting attending and associated residents would cover that procedure. Following completion of all cases requiring a postoperative admission, all orders were placed by the operating resident or attending and the patients were signed out to the on-call team.

A separate resident team and attending were assigned to the Trauma and Trauma/Surgery ICU as shown in Table 2. One attending, 1 postgraduate year (PGY)-4 resident, 1 PGY1 intern, and 3 rotating midlevel providers were assigned to the trauma service to care for all patients admitted to trauma service on the inpatient wards. They were not responsible for new patient consultations or trauma code activations. The Trauma/Surgery Critical Care service was staffed by a board-certified critical care attending, 1 PGY2 resident, and 2 rotating midlevel providers. The senior resident on each of these services coordinated with attending and midlevel providers to arrange for daily coverage allow for absences and days off in keeping with the Accreditation Council for Graduate Medical Education (ACGME) standards for trainee duty hour restrictions.^3^

The Pediatric Surgery service was limited to EC and inpatient consultations. Elective cases were avoided when possible. Given the reduced patient volume and case load, a single PGY3 resident and 2 rotating midlevel providers were assigned to 2 pediatric surgeons on this service on an as-needed basis. The resident and midlevel providers were responsible for daily inpatient rounds and scheduled cases. The resident was excused from outpatient office coverage. The resident was then allowed to take home-call upon completion of all in-house duties.

Similarly, as the patients on the surgical oncology service were determined to be an at-risk population for virus transmission/contraction, outpatient office resident coverage was discontinued. Encounters were converted to virtual platforms and a single PGY3 resident was assigned to assist with the reduced volume of inpatients and scheduled cases. This resident coordinated with the on-call senior resident for inpatient round coverage to allow for minimum 1 day off each week. The vascular surgery service was staffed by 4 vascular surgeons each with their own midlevel provider, 1 surgical first assistant, and 1 PGY5 resident.

The micro-teams staffed by surgical intensivists were positioned in a cascading fashion to cover the additional COVID ICUs as they were sequentially activated. The junior and senior level residents assigned to these attendings were deployed with their attending to the COVID care unit.

### Backup Coverage

Given the markedly reduced number of residents in house, a system for backup coverage was adopted such that if the decision was made that additional personnel were needed, then the attending on call would communicate this need to the backup attending who would then activate their team. A backup call schedule is generated monthly and the associated teams were made aware of their responsibility during that period. Additionally, a rotating 3-night-on/3-night-off night float system was instituted rotating available PGY2 residents to assist with nocturnal Surgical/Trauma ICU service coverage and new patient consults as indicated.

### Educational Adjustments

Meeting in groups greater than 10 was discouraged forcing an adjustment to ensure teaching conferences and educational initiatives could be met. Virtual platforms were quickly adopted to continue weekly resident education conferences led by a rotating senior resident and junior resident supervised by the Associate Chair for Clinical Education. This weekly conference was augmented with preconference and post-conference quizzes assigned to each resident via the Surgical Council on Resident Education (SCORE®) portal with the results transmitted to the program director and administrative resident for review. Attendance at the weekly teaching conference was mandatory for all PGY1-4 residents unless excused for emergency circumstances. The PGY5 residents were required to independently complete modules in the Pass Machine curriculum; their progress was monitored separately by the program director. PGY4-5 residents underwent mock oral examination during this time along with interdepartmental individual assessment of leadership, clinical capacity, and performance.

Grand Rounds and other didactic opportunities such as Gastrointestinal Conference, Tumor Board, and Trauma System meetings were canceled until virtual platforms could be established. It was decided that confidential meetings such as morbidity and mortality for general surgery and trauma should not be held through the digital platforms at this time. However, small group assemblies, socially distanced, with the trauma director and performance improvement coordinator were carried out to ensure quality, and patient safety measures were met and improvement opportunities were addressed.

In accordance with guidelines published by the American Association of Medical Colleges (AAMC), medical students were excused from direct patient care duties until sufficient PPE could be acquired by the hospital system.^4,5^ The clerkship directors and administration from Mercer made arrangements for all students in the midst of their clerkships to adopt a virtual didactic curriculum for approximately 8 weeks. During that time the resource availability was assessed and sufficient PPE was secured to allow medical students to resume their clinical rotation experiences. Abbreviated clerkship rotations were developed with emphasis placed on clinical decision-making and direct patient care when appropriate.

### Wellness

Given the significant mental and physical stress associated with the provision of care during this time, the senior surgery residents made themselves available to the junior residents for informal virtual “debriefing sessions” and “happy-hours” through available audio and video platforms. These sessions remain informal and are not recorded. These sessions have been encouraged by department leadership, which asked all residents to conduct themselves responsibly and observe social distancing during this time. Additionally, all members of department leadership were available should a resident desire to address specific personal, patient, or system-related concerns.

## Surge Plan

**Figure 1.**
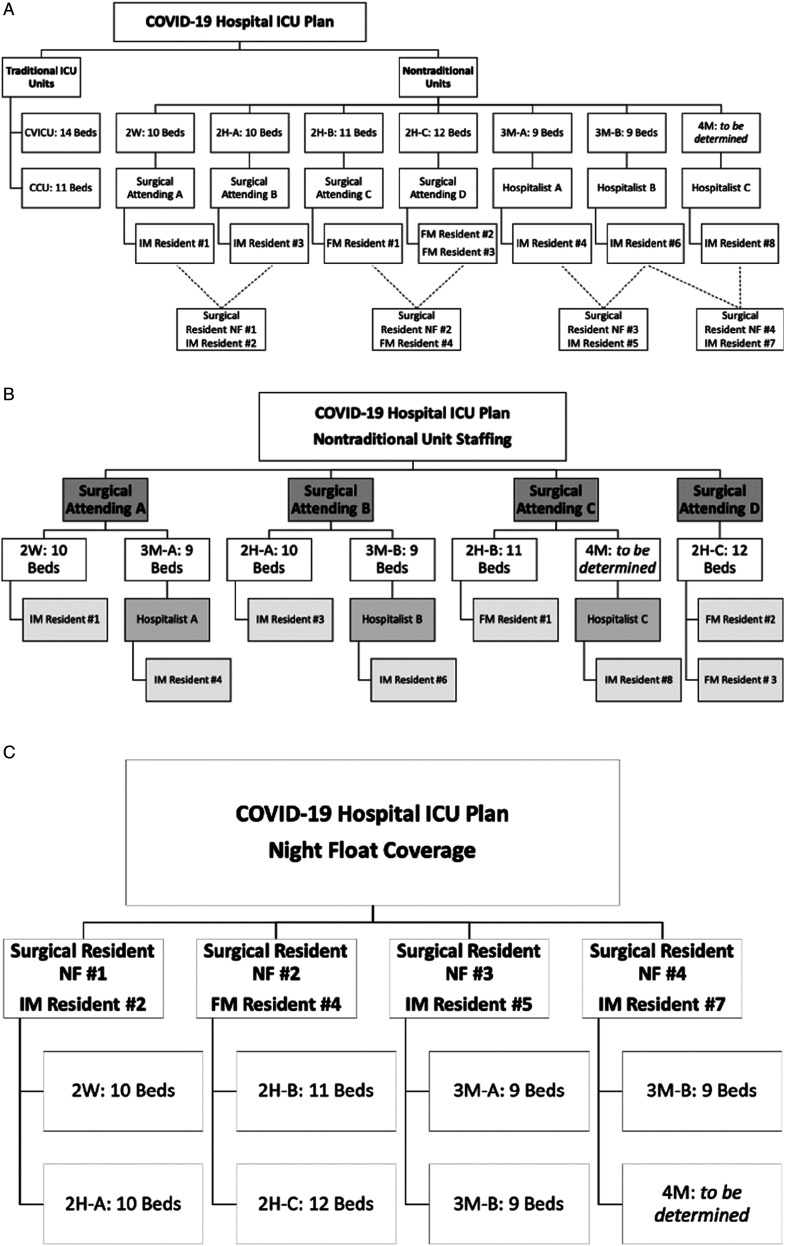
COVID-19 Hospital ICU Plan. (A) Overview of traditional and nontraditional intensive care units developed and staffing assignments to nontraditional ICU units. The Cardiovascular Intensive Care Unit and Critical Care Unit had no change in intensivist assignment, staffing, and management. Surgical attendings A-D are credentialed for critical care. The surgical attendings’ team consisted of the assigned surgical residents (Table 1) and an internal/family medicine resident. Hospitalists were provided from staff medicine attendings and contracted hospitalists. (B) Management of the nontraditional ICUs was modeled after the Society of Critical Care Medicine’s Tiered Staffing Strategy.^6^ Once the patient load required for activation and use 3M-A for patient care, surgical intensivists would cover 2 units in our modified tiered staffing strategy. A surgical attending credentialed in critical care would oversee the nontraditional units and a non-ICU physician (Hospitalists A-C) would work alongside the surgical attending. This would maximize care for the large number of COVID-19 patients in critical condition. (C) Night float assignment for the nontraditional units. The units were covered by a surgery resident and an internal/family medicine resident. ICU, Intensive care unit.

Once the general surgery service plan was developed, the surgical critical care service was asked to participate in the development of the institution wide intensive care surge plan. The charge was to provide plans for physician staffing of up to 190 critical care beds. Areas throughout the hospital that had the appropriate infrastructure were identified for nontraditional ICU units, and a staffing model that involved participation from all residencies, medical and surgical intensivists, and hospitalists was developed as depicted in Figure 1. Each additional nontraditional ICU was composed of a surgical intensivist, 2 surgery residents, and 2 internal medicine or family medicine residents. The night float resident would cover 2 units while on call. These units were to be activated in a sequential fashion as needed with surgical intensivists A, B, C, and D, respectively. The next 3 units to be activated would have the addition of a hospitalist to the resident team as the surgical intensivist would then be responsible for covering 2 units. Although this is not optimal, it is certainly feasible in a disaster surge plan and is consistent with the Society of Critical Care Medicine recommendations for COVID-19 coverage.^6^ Residents from pediatrics and obstetrics and gynecology (OB/GYN) were assigned to a flexible reserve pool and remained available as needed.

## Discussion

Restructuring the general surgery residency allowed for continued medical care and education while reducing exposure risk to surgical residents and faculty. The collaboration between residency programs and hospital physician surge planning committee allowed the institution to develop a hospital-wide ICU surge plan. This plan doubled the ICU capacity from approximately 80 ICU beds to a potential 190 ICU beds. While there is no “1 size fits all” model or plan for any type of disaster scenario, our tiered, micro-team approach to coverage insulated the workforce, allowed maximal flexibility, and minimal exposure risk all while maintaining high clinical performance standards. Our strategy is scalable in that residents, and midlevel providers can be added to service lines to further improve flexibility to accommodate potential escalating ICU duties or fill workforce gaps if infectious exposures were to occur.

Emergency response and preparedness for rapid patient influx is not wholly unfamiliar to surgeons. Indeed, emergency responses to mass casualty events are frequently discussed and practiced at trauma centers on nationwide scale in preparation for events such as shootings, bombings, or natural disasters. In many instances, the underlying principles of a mass casualty response and the systemic response to the current pandemic are similar. Communication, teamwork, patient triage strategies, assessments of supply chains, and resource utilization models are common concepts to both the mass casualty event and the patient surges seen with the COVID-19 pandemic. Additionally, both occurrences are known to physically and mentally challenge a workforce. However, the contagious nature of COVID-19 creates further challenges when considering protecting our workforce from infection, exhaustion, and burnout.^7^

Our experience provides a potential usable model for future responses to surges related to infectious diseases and other crises requiring multiple small teams to function in concert independently. The utilization of a team-based approach limits high-risk exposures, addresses mental and physical health concerns, complies with local and national guidelines and recommendations, propagates medical education, and ensures continued high-quality patient care.
